# Disparities in access to eating disorders treatment for publicly-insured youth and youth of color: a retrospective cohort study

**DOI:** 10.1186/s40337-022-00730-7

**Published:** 2023-01-24

**Authors:** Ruby Moreno, Sara M. Buckelew, Erin C. Accurso, Marissa Raymond-Flesch

**Affiliations:** 1 Department of Pediatrics, University of California, San Francisco, San Francisco, CA USA; 2 Division of Adolescent & Young Adult Medicine, University of California, San Francisco, San Francisco, CA USA; 3 Department of Psychiatry and Behavioral Sciences, UCSF Weill Institute for Neurosciences, University of California, San Francisco, San Francisco, CA USA; 4 Philip R. Lee Institute for Health Policy Studies, University of California, San Francisco, San Francisco, CA USA

**Keywords:** Family-based treatment (FBT), Cognitive behavioral therapy (CBT), Anorexia nervosa (AN), Atypical anorexia nervosa (AAN), Bulimia nervosa (BN), Binge eating disorder (BED), Public insurance, County rurality, Structural racism, Hospitalization

## Abstract

**Background:**

Eating disorders are associated with substantial morbidity and mortality that can be minimized by timely access to evidence-based treatment. However, disparate access to eating disorders treatment may contribute to significant health disparities amongst marginalized groups. This study examined the association between insurance type (public vs. private) and receipt of recommended mental health treatment in a sample of racially/ethnically diverse youth who presented to an adolescent medicine clinic with malnutrition secondary to disordered eating.

**Methods:**

A retrospective chart review was conducted for youth ages 11–25 years (*N* = 1060) who presented to an urban adolescent medicine specialty program between June 1, 2012 and December 31, 2019 for malnutrition secondary to disordered eating. Bivariate and logistic regression analyses examined the association between insurance type (public vs. private) and other demographic/clinical factors on receipt of recommended treatment within six months of the initial evaluation.

**Results:**

Patients with public insurance were one third as likely to receive recommended treatment as patients with private insurance (AOR = 3.23; 95% CI = 1.99, 4.52), after adjusting for demographic and clinical factors. Latinx (AOR = 0.49; 95% CI = 0.31, 0.77) and Asian (AOR = 0.55; 95% CI = 0.32, 0.94) patients were half as likely to receive recommended treatment as White patients.

**Conclusions:**

Access to evidence-based mental health treatment is a necessary first step towards health equity for individuals with eating disorders. Additional work is needed to dismantle systemic inequities that contribute to disparities in care for youth of color and those with public insurance.

**Supplementary Information:**

The online version contains supplementary material available at 10.1186/s40337-022-00730-7.

## Plain English Summary

Early management of eating disorders, including mental health and medical treatment, improves outcomes and reduces the likelihood of significant complications including death. However, access to specialized mental health treatment can be challenging, particularly for patients from marginalized identities. This study examined how insurance type affects access to recommended mental health treatment for patients with eating disorders. It included a sample of 1060 youth of diverse racial/ethnic backgrounds who presented to an adolescent medicine clinic with malnutrition secondary to disordered eating. In our sample, youth with public insurance were one third as likely to receive appropriate treatment as youth with private insurance. Additionally, Latinx and Asian patients were half as likely to receive appropriate treatment as White youth. These findings highlight the importance of not only improving access to mental health care for patients with eating disorders, but also addressing the systemic causes of disparities in care for youth of color and those with public insurance.

**Supplementary Information:**

The online version contains supplementary material available at 10.1186/s40337-022-00730-7.

## Background

Eating disorders are associated with one of the highest mortality rates among psychiatric conditions [1–3]. Youth are disproportionately affected, with higher observed mortality rates among people ages 15 to 29 [4]. Prognosis for adolescents and young adults with eating disorders is improved with early mental health treatment and medical care [5]. However, differential access to these services could contribute to disparities in outcomes, including higher rates of suicidality and cardiovascular, pulmonary, metabolic, and gastrointestinal disease [6].

Recent studies demonstrate that people who are racial and ethnic minorities^1^ in the United States (U.S.), referred to hereafter as people of color, receive mental health care at lower rates despite similar eating disorder prevalence rates [7, 8]. Youth of color with documented mental health concerns are about half as likely to receive mental health care as their White peers [9]. Research also shows eating disorder behavior prevalence rising at a faster rate for individuals of lower socioeconomic status compared to individuals of higher socioeconomic status [10]. While information on services available to youth with public insurance is limited, evidence suggests that public insurance may be a barrier to accessing specialized eating disorder treatment [11, 12]. In fact, youth with public insurance who have restrictive eating disorders have been described as grossly underserved due to under-resourced health care systems that are unable to meet patient needs [13].

There are nearly 37.4 million children enrolled in Medicaid and the Children’s Health insurance Program (CHIP) in the U.S. today [14]. Children of color, who now comprise more than half of the US children’s population, [15] are overrepresented in these programs [16] because they are more likely to live in poverty [17]. With youth at the forefront of the changing demographic profile in the U.S., understanding access to mental health care for diverse and publicly-insured youth is imperative.

To our knowledge, the specific impact of insurance on access to care for racially and ethnically diverse youth with eating disorders has not been studied. The objective of this study is to describe the association between racial and ethnic minority status and insurance type (public vs. private) on access to recommended eating disorders treatment for youth who presented for outpatient medical care with malnutrition secondary to disordered eating. We address this objective with a retrospective chart review of adolescents and young adults presenting to a specialized outpatient eating disorders program within an adolescent medicine practice. We hypothesized that public insurance would be associated with lower odds of receiving the recommended treatment, even after adjusting for demographic and clinical characteristics.

## Methods

We conducted a retrospective chart review of eating disorder patients ages 11–25 years with malnutrition secondary to disordered eating who presented to an urban academic adolescent medicine clinic for a medical intake visit between June 1, 2012 and December 31, 2019. The program includes five urban and suburban medical clinics in Northern California that provide full-spectrum interdisciplinary care for adolescents and young adults, including primary care, sexual and reproductive health care, and medical management of mental health disorders, including eating disorders. At the time of this study, the clinic was serving approximately 1500 unique patients per year with a catchment area encompassing 42 of California's 58 counties. Institutional Review Board (IRB) approval was obtained at the medical institution associated with the program.

Charts were identified by query of the electronic medical record (Epic Systems). Patients whose diagnosis or reason for visit was “malnutrition”, “eating disorder”, or “disordered eating” at a new visit within the adolescent medicine department during the study period were identified. We used the medical diagnosis of malnutrition to identify patients because the data were drawn from a medical clinic population for which malnutrition is the most common indication for referral and treatment. Patients with a diagnosis of malnutrition unrelated to disordered eating (e.g., due to a medical condition) were excluded through manual review. Date of the new patient visit was obtained and confirmed to be a new Eating Disorders Program intake visit through manual review. The initial query yielded 1375 new eating disorder visits during our study period. After manual exclusion of those who did not meet inclusion criteria, our final sample size was 1060. The following data were collected from deidentified charts: (1) demographics, (2) hospital admissions, (3) DSM-5 diagnosis (if applicable), (4) treatment recommended by the assessing clinician, and (5) type of treatment received.

Treatment recommendations were made by the assessing clinician (specialized mental health and/or Adolescent Medicine providers) upon initial evaluation within the eating disorders program. Treatment recommendations are highly standardized within the program and consistent with expert guidelines for adolescents and adults [18–21]. In addition, these recommendations are based on clinical consensus following presentation and discussion of the diagnosis and treatment considerations in weekly team meetings. Adolescents with restrictive eating disorders almost always received referrals for family-based treatment (FBT) except when contraindicated (e.g., family history of abuse, significant borderline personality disorder features, prior failure of evidence-based outpatient treatments), in which case adolescent-focused therapy (AFT), cognitive behavioral therapy (CBT), dialectical behavior therapy (DBT), or higher level of care were considered. For adolescents with avoidant restrictive food intake disorder or binge-purge type disorders, FBT and CBT were both considered given limited data suggesting that both are effective. Cognitive behavioral therapy (CBT) was often the first-line recommendation for adults with eating disorders, unless they were living at home and preferred FBT, or a higher level of care was indicated [22].

### Primary outcome

#### Recommended treatment

The primary outcome measure in this study was whether a patient received the treatment that was recommended by the expert clinician within six months of intake (see Additional file 1: Table S1 for list of diagnoses and therapies received by coding). Treatment recommendations were obtained from the encounter documentation (assessment and plan section of the note), which is standardly included in the note template for intake visits. In cases where the recommended treatment was not clearly documented in the assessment and plan section of the notes (< 5% of cases), the chart was reviewed by the Clinical Director of the Eating Disorders Program (ECA), who made an assessment about the recommended therapy based on the clinic's best practices for therapy recommendations at the time. Standard documentation for follow-up visits included whether or not a patient was engaged in therapy and if so, what type of therapy. Charts were coded as to whether participants had received the recommended treatment within six months of evaluation. Patients with no follow-up visits within six months were considered lost to follow-up, as all patients are recommended to have at least one follow-up visit with the program prior to discharge from the clinic.

### Covariates

#### Demographics

Demographic characteristics were abstracted from the chart, including age, gender identity (female, male, or non-binary), racial and ethnic^2^ (herein referred to as racial/ethnic) minority status, language (English, non-English), primary medical insurance (private, public, self-pay, none), and county of residence. U.S. Census 2010 county rural lookup tables were used to identify rural percentage for each county [23].

#### Diagnosis

Eating disorder diagnosis was obtained from clinical notes and recoded according to the DSM-5. Diagnoses were collapsed into the following categories for analysis: Anorexia Nervosa (AN), Atypical Anorexia Nervosa (AAN), Avoidant Restrictive Food Intake Disorder (ARFID), BN (Bulimia Nervosa), and Other, which included Binge Eating Disorder (BED), Other Specified Feeding or Eating Disorder (OSFED), Rumination disorder, and Unspecified Feeding or Eating Disorder (UFED). Patients with eating disorder symptoms not meeting criteria for an eating disorder diagnosis were excluded from analyses.

#### Hospitalization

Recent (30 days prior to or on the date of intake) hospitalization for medical instability secondary to disordered eating was obtained through chart review as a marker of medical severity at presentation.

### Statistical analysis

Bivariate analyses (Chi-square tests, t-tests) were used to describe demographic and clinical differences in patients by insurance type. Bivariate analyses were also used to identify demographic and clinical factors that predicted receipt of the recommended treatment (yes/no) within six months of the initial medical evaluation. Multivariate logistic regression analyses were then performed to assess for the independent effect of demographic and clinical factors on receiving the recommended treatment. The first step in the regression included age, gender, racial/ethnic minority status, insurance type, county rurality, diagnosis, and hospitalization. The second step included significant factors from the first step and interactions between race/ethnicity and insurance type. Reference categories were used for categorical variables. Due to the correlation of preferred language with race/ethnicity and insurance type, a separate analysis was performed in a subset of patients (Latinx with public insurance) to assess for the impact of language on receipt of recommended treatment. This group was selected for sub analysis, as the vast majority of patients in our sample who indicated a primary language other than English were Spanish-speaking. This multivariate logistic regression included language and significant main effects from step one of the primary multivariate model. Missing values were excluded from analyses. Bivariate analyses were performed for predictor variables in patients missing our primary outcome variable (therapy type) due to loss to follow up. All analyses were conducted with IBM SPSS Statistics (version 27).

## Results

Our sample consisted of 1060 adolescents and young adults presenting to an urban adolescent medicine specialty program for monitoring and medical management of eating disorders with a mean age of 16 years; most were female (85.7%) and about half (54.5%) were White (Table 1). Nearly all patients were insured (75.9% private coverage, 23.5% public coverage). Patients with no insurance (*n* = 2) or who did not use their insurance for services (self-pay, *n* = 4) were excluded from analysis due to small sample size. Participants lost to follow-up with missing data were more likely to have an “other” eating disorder diagnosis and no history of hospitalization. Missing data were not associated with any other main predictors.

**Table 1:** Characteristics of ED patients evaluated, by insurance type

	Total (*N* = 1,060), % (n) or Mean (SD)	Private (*N* = 805), % (n) or Mean (SD)	Public (*N* = 249), % (n) or Mean (SD)	*p*
Age	16.0 (3.0)	16.2 (3.1)	15.5 (2.6)	< 0.001
Gender				0.001
Female	85.7% (908)	87.6% (705)	79.1% (197)	
Male	13.0% (138)	11.6% (93)	18.1% (45)	
Non-Binary	1.3% (14)	0.9% (7)	2.8% (7)	
Race/ethnicity				< 0.001
Asian	7.5% (80)	8.2% (66)	5.6% (14)	
Black/African American	2.2% (23)	1.2% (10)	5.2% (13)	
Latinx	17.7% (188)	7.3% (59)	51.4% (128)	
Other	8.6% (91)	8.9% (72)	7.6% (19)	
Native American/Alaskan Native	0.5% (5)	0.6% (5)	0% (0)	
Native Hawaiian/Other Pacific Islander	0.4% (4)	0.5% (4)	0% (0)	
Other race	7.7% (82)	7.8% (63)	7.6% (19)	
White	54.5% (578)	64.3% (518)	22.5% (56)	
Unknown	9.4% (100)	9.9% (80)	7.6% (19)	
Language				< 0.001
English	92.5% (978)	99.0% (794)	71.9% (179)	
Non-English	7.5% (79)	1.0% (8)	28.1% (70)	
Chinese	0.6% (6)	0.2% (2)	1.6% (4)	
Spanish	6.6% (70)	0.7% (6)	25.7% (64)	
Other	0.3% (3)	0% (0)	0.8% (2)	
County Rurality	3.5 (0.3)	3.2 (6.4)	4.6 (10.2)	0.04
Diagnosis				0.06
AN	38.4% (407)	39.3% (316)	35.7% (89)	
AAN	13.3% (141)	13.0% (105)	14.1% (35)	
ARFID	6.1% (65)	6.3% (51)	5.2% (13)	
BED	1.6% (17)	2.0% (16)	0.4% (1)	
BN	6.1% (65)	6.6% (53)	4.8% (12)	
UFED	27.6% (293)	25.6% (206)	34.1% (85)	
Other specified eating disorders	5.5% (58)	6.1% (49)	3.6% (9)	
ED symptoms not meeting diagnostic threshold	1.3% (14)	1.1% (9)	2.0% (5)	
Prior hospitalization	22.2% (235)	21.6% (174)	24.5% (61)	0.34
Lost to follow-up	14.0% (148)	13.2% (106)	16.1% (40)	0.25

ED = eating disorder; AN = anorexia nervosa; AAN = atypical anorexia nervosa; ARFID = avoidant restrictive food intake disorder; BED = binge eating disorder; BN = bulimia nervosa; UFED = unspecified feeding or eating disorder

*p* values are for result of t-test or chi-square analysis

Other specified eating disorders includes Other Specified Feeding or Eating Disorders (OSFED) with specifiers and Rumination disorder

Compared to patients with private insurance, those with public insurance presented at a significantly younger age (public 15.5 years vs. private 16.2 years, *p* < 0.001) and were more likely to be male (18.1% vs. 11.6%, *p* = 0.001) or non-binary (2.8% vs. 0.9%, *p* = 0.001). They were also significantly more likely to be Latinx (51.4% vs. 7.3%, *p* < 0.001) or Black (5.2% vs. 1.2%, *p* < 0.001) and endorse a language other than English as their preferred language (28.1% vs. 1.0%, *p* < 0.001; Spanish: 25.7%, Chinese: 1.6%, other: 0.8%). There were no significant differences seen in diagnoses, hospitalization, county rurality, or loss to follow up between the public and private insurance samples.

Of those with follow-up data (86.0%, *n* = 912), 39.4% (*n* = 359) of youth did not receive the recommended treatment, with 22.6% (*n* = 206) who received a treatment other than what was recommended and 16.8% (*n* = 153) who received no treatment despite treatment having been recommended. For youth to whom treatment was recommended, race/ethnicity was significantly associated with failure to receive any treatment (Χ^2^ = 13.298, *p* = 0.021), with Latinx individuals being more likely to receive no treatment (21.3%) than Whites (11.4%), with no differences in receipt of treatment for individuals whose race was Asian, Black/African American, Other, or Unknown. Failure to receive any treatment was also more common for those with public insurance (20.1%) versus private insurance (12.8%) (Χ^2^ = 8.134, *p* = 0.004). Failure to receive any treatment was also more common for males (22.5%) than females (13.3%) (Χ^2^ = 8.710, *p* = 0.013), with no differences for non-binary youth. There were no differences by age (*p* = 0.60). Factors associated with receipt of the recommended treatment in bivariate analysis (Table 2) included race/ethnicity (Asian, Latinx, Black, White, and Other), language, insurance type, diagnosis, and hospitalization.

**Table 2:** Bivariate analysis of factors associated with recommended treatment

	Received recommended treatment				
	Yes (*n* = 553)	No (*n* = 359)	χ^2^	df	*p*
	% (n)	% (n)			
	or Mean (SD)	or Mean (SD)			
Age	15.9 (3.1)	16.0 (2.7)			0.47
Gender			3.75	2	0.15
Female	87.3% (483)	83.3% (299)			
Male	11.8% (65)	14.8% (53)			
Non-Binary	0.9% (5)	1.9% (7)			
Race/ethnicity			53.28	5	< 0.001
Asian	7.9% (39)	10.6% (35)			
Latinx	10.6% (52)	29.6% (97)			
Black/African American	1.6% (8)	3.7% (12)			
Other	8.7% (43)	9.1% (30)			
White	71.1% (350)	47.0% (154)			
Language			40.49	1	< 0.001
English	96.9% (534)	85.5% (306)			
Non-English	3.1% (17)	14.5% (52)			
Insurance Type			69.28	1	< 0.001
Private	86.4% (475)	62.6% (224)			
Public	13.6% (75)	37.4% (134)			
County rurality	3.3 (7.0)	3.4 (7.6)			0.87
Diagnosis			48.48	4	< 0.001
AN	48.0% (264)	30.1% (107)			
AAN	15.3% (84)	13.0% (46)			
ARFID	5.8% (32)	6.5% (23)			
BN	6.7% (37)	5.1% (18)			
Other	24.2% (133)	45.4% (161)			
Prior hospitalization					
No	70.9% (392)	84.1% (302)	20.97	1	< 0.001
Yes	29.1% (161)	15.9% (57)			

AN = anorexia nervosa; AAN = atypical anorexia nervosa; ARFID = avoidant restrictive food intake disorder; BN = bulimia nervosa

Other race/ethnicity includes Native American/Alaskan Native, Native Hawaiian/Other Pacific Islander, and other race

Other diagnosis includes Binge Eating Disorder (BED), Unspecified Feeding or Eating Disorder (UFED), Other Specified Feeding or Eating Disorders (OSFED) with specifiers, and Rumination disorder

The multivariate analysis (Table 3) found that patients with private insurance were three times more likely to receive recommended treatment than patients with public insurance (AOR = 0.31; 95% CI = 0.17, 0.59, *p* < 0.001). In the same model, racial and ethnic minority youth were less than two thirds as likely to receive recommended treatment compared to White youth (AOR = 0.63; 95% CI = 0.45, 0.89, *p* = 0.008). Patients who were hospitalized were nearly twice as likely to receive recommended treatment as those who were not (AOR = 1.96; 95% CI = 1.32, 2.94, *p* < 0.001). Patients diagnosed with “other" eating disorders were half as likely to receive recommended treatment when compared to AN (AOR = 0.41; 95% CI = 0.28, 0.59, *p* < 0.001)). The interaction between racial/ethnic minority status and insurance type was not significant (*p* = 0.68).

**Table 3:** Logistic regression of factors associated with recommended treatment

	Step 1		Step 2	
	AOR	95% CI	AOR	95% CI
Age	0.95	0.90, 1.00		
Gender				
Female (reference)				
Male	0.85	0.53, 1.36		
Non-Binary	0.93	0.25, 3.43		
Race/ethnicity*				
White (reference)	0.55*		0.63**	0.45, 0.89
Racial or ethnic minority		0.32, 0.94		
Insurance type*				
Private (reference)	0.30***	0.20, 0.45	0.31***	
Public				0.17, 0.59
County rurality	1.00	0.98, 1.02		
Diagnosis*				
AN (reference)				
AAN	0.83	0.52, 1.34	0.83	0.53, 1.30
ARFID	0.53	0.27, 1.04	0.59	0.32, 1.10
BN	1.07	0.54, 2.15	0.97	0.51, 1.85
Other	0.41***	0.28, 0.60	0.38***	0.27, 0.54
Prior hospitalization*				
No (reference)	1.92**	1.28, 2.85	1.92***	1.32, 2.51
Yes				
Race/ethnicity * insurance				
White × private (reference)			0.85	0.40, 1.83
Minority × public insurance				

AOR = adjusted odds ratio; CI = confidence interval, AN = anorexia nervosa; AAN = atypical anorexia nervosa; ARFID = avoidant restrictive food intake disorder; BN = bulimia nervosa

Other diagnosis includes Binge Eating Disorder (BED), Unspecified Feeding or Eating Disorder (UFED), Other Specified Feeding or Eating Disorders (OSFED) with specifiers, and Rumination disorder

Step 1: Age, gender, race/ethnicity, insurance type, county rurality, diagnosis, hospitalization

Step 2: Age, race/ethnicity, insurance type, diagnosis, hospitalization, race/ethnicity*insurance

*p ≤ 0.05; **p ≤ 0.01; ***p ≤ 0.001

In the sub analysis to assess the impact of language on receipt of the recommended treatment among publicly-insured Latinx youth (Table 4), language was not a significant predictor. However, Latinx patients who were hospitalized were more than four times more likely to receive recommended treatment than those who were not (AOR = 4.33; 95% CI 1.52, 12.20, *p* = 0.006), after adjusting for eating disorder diagnosis. In addition, patients with AN were 14 times more likely than patients with AAN to receive the recommended treatment (AOR = 0.07; 95% CI 0.01, 0.62, *p* = 0.017), while those with “other” eating disorder diagnoses were one third as likely to receive recommended treatment (AOR = 0.31; 95% CI 0.10, 0.99, *p* = 0.048).

**Table 4:** Logistic regression of factors associated with receipt of recommended treatment among Latinx patients with public insurance

	AOR	95% CI
Language		
English (reference)	0.84	0.31, 2.24
Non-English		
Diagnosis		
AN (reference)		
AAN	0.07*	0.01, 0.62
ARFID	2.29	0.27, 19.26
BN	0.69	0.11, 4.35
Other	0.31*	0.10, 0.99
Prior hospitalization		
No (reference)		
Yes	4.33**	1.52, 12.20

AOR = adjusted odds ratio; CI = confidence interval, AN = anorexia nervosa; AAN = atypical anorexia nervosa; ARFID = avoidant restrictive food intake disorder; BN = bulimia nervosa

Other diagnosis includes Binge Eating Disorder (BED), Unspecified Feeding or Eating Disorder (UFED), Other Specified Feeding or Eating Disorders (OSFED) with specifiers, and Rumination disorder

*p ≤ 0.05; **p ≤ 0.01

## Discussion

Although eating disorders are equally prevalent across racial and socioeconomic groups, [7, 8, 10] there are limited data on the extent to which eating disorders are appropriately identified and treated among marginalized groups. In this study, we found that about 40% of youth with eating disorders did not receive the treatment recommended to them based on expert clinical treatment guidelines. Youth with eating disorders who had public insurance were significantly more racially and ethnically diverse than those with private insurance, and both factors (i.e., public insurance and race/ethnicity) were independent barriers to receiving the recommended eating disorders treatment. We also found that patients admitted to the hospital upon medical evaluation were significantly more likely to receive recommended treatment than those who were not admitted. These results highlight multiple structural barriers to care experienced by diverse youth with eating disorders.

As we hypothesized, insurance type independently predicted therapy received in our sample. Youth with public insurance had one third the odds of receiving recommended treatment compared to those with private insurance. While insurance coverage is a known barrier to intensive outpatient and residential eating disorders treatment, [11, 24] patients with public insurance may face additional barriers to eating disorders care, including finding a provider trained in evidence-based treatments for eating disorders. Family-based treatment (FBT) and cognitive behavioral therapy (CBT) are often the most effective treatments for adolescents with eating disorders, [25, 26] with FBT being the only psychosocial treatment currently meeting criteria as a Level 1 (Well Established) treatment for adolescents with AN and leading to significantly faster improvement in outcomes, fewer days in hospital due to medical complications, and better long-term outcomes [27–31]. Further, specialized outpatient treatment is significantly more cost-effective than generalist outpatient treatment [32]. However, access to the limited number of clinicians trained in evidence-based treatments is challenging, particularly in the public sector [26, 33, 34]. In our program, many publicly-insured patients are not able to access FBT at our institution or in the community, instead relying on therapists with limited eating disorder experience who provided non-specialized individual therapy. This places youth with eating disorders at further risk of medical complications and directly contributes to disparities in care. While there are limited data on treatment outcomes for publicly-insured youth, recent trends show a higher increase in eating disorder hospitalizations paid for by Medicaid compared to private payors [35]. These findings point towards the need for policy and/or funding changes to improve access to mental health care for eating disorders among publicly-insured youth.

Further, our study showed that after adjusting for clinical and demographic characteristics including insurance type, youth of color are still less than two thirds as likely to receive recommended treatment compared with White youth. This finding is consistent with national data about mental health care access for youth of color with other mental health diagnoses [9]. It highlights the need to examine the role of structural racism [36] in treatment of eating disorders. For example, there is a growing body of literature in other patient populations demonstrating that patient-provider racial and ethnic concordance may improve care through improving patient-reported satisfaction with care [37] and improving a patient’s working alliance with mental health providers [38]. However, it is well documented that Latinx and Black providers are underrepresented in medicine and mental health care [39, 40]. It is also possible that youth of color live in communities with fewer specialized mental health providers [9]. Additional research is needed to identify which of these factors might be impacting access to appropriate therapy for youth with eating disorders.

Outside of this study, provider bias is another structural factor that impacts mental health referrals. Although eating disorders in people of color, males, and non-binary identifying individuals are increasingly being recognized, [41, 42] for many years eating disorders were believed to occur predominantly in White female patients [43]. The effects of this bias on outcomes for youth of color with eating disorders are far reaching. While studies have shown that people of color are less likely to be screened for or receive an eating disorder diagnosis [7, 44] our study also indicates that youth of color—when identified—are less likely to receive recommended treatment. While race and insurance type are undoubtedly correlated in our healthcare system [16] and in our sample, these factors were independently associated with therapy outcome, demonstrating that insurance coverage alone cannot guarantee equity in the treatment of eating disorders and suggestive of more pervasive systemic inequity within the healthcare system.

Stigma and cultural beliefs about mental health care may contribute as additional barriers to eating disorder care for youth of color, [45] but data on the systemic barriers to care are lacking, in part, due to the historical under-recognition of eating disorders in this population [46]. As providers, it is our role to examine and address the systemic barriers that contribute to disparities in care for racially and ethnically diverse patients, and these findings highlight the need for further targeted interventions for racially/ethnically marginalized groups.

Among Latinx patients with public insurance specifically, those with AAN were significantly less likely to receive recommended treatment than those with AN. As there is no evidence that the morbidity of AAN is any lower than that of full-threshold AN, [47] barriers in access to therapy for this population could result in worse outcomes. Further, publicly-insured Latinx patients were less likely to receive recommended treatment. This is an important consideration in providing equitable eating disorder treatment to Latinx youth, as the Latinx rural population continues to grow [48].

Finally, across all models, a hospital admission for disordered eating within 30 days of intake significantly increased the likelihood of receiving recommended treatment. This has important implications for the accessibility of eating disorders treatment. While a hospital admission may be necessary for medical stabilization and transfer to an appropriate level of care, it is both costly and dangerous as a necessary pathway to treatment. Although our study did not evaluate aspects of hospitalization that may impact receipt of care, most hospitalized patients in our study underwent comprehensive evaluation by a multidisciplinary team, including psychology, social work, and nutrition, who often advocated to behavioral health systems on behalf of patients. Upon stabilization, patients were typically discharged to recommended treatment and scheduled for medical follow-up. Our finding points to a structural barrier to care for eating disorder patients that necessitates further research.

### Limitations

There are several limitations to this study. First, this was a retrospective chart review conducted at single urban academic specialty program. Our sample focused on youth with malnutrition secondary to disordered eating which excluded patients without weight loss and/or those who had not fallen off their growth curves, including many patients with BED. Therefore, our findings may lack generalizability to the larger population of youth with eating disorders, where binge eating disorder is much more common [49], and certain analyses should be interpreted with caution due to the retrospective nature of data collection (e.g., preferred language was inconsistently documented). Additionally, due to our program’s academic nature, some of our participants received evidence-based therapy at no cost through participation in a federally-funded treatment trial with active recruitment over two years during the study period. Our results therefore likely underestimate the impact of public insurance on therapy type received, as many publicly-insured patients would not have otherwise had access to FBT. Second, there are limitations to the interpretation of race/ethnicity in our study. Specifically, we recognize that race is a social construct [50] and that the categories used in this study are imperfect measures of people’s identities, lived experiences, and experiences of racism. Nevertheless, we felt it important to include race in our analyses to investigate the contribution of racial/ethnic minority status to disparities in treatment access, after adjusting for other measured factors, given increased understanding about how structural racism negatively impacts health outcomes for immigrants as well as racial and ethnic minorities [51–53]. Third, Black patients were notably under-represented given the racial/ethnic demographics in California (2.2 vs 5.4%, respectively) [53]. Although the public insurance group was more representative of California’s Black youth (5.2%), the overall small number of Black patients in our sample did not allow for meaningful analysis of outcomes for Black youth. Their under-representation could itself suggest structural racism in the diagnosis, referral, and treatment of patients with eating disorders. Moreover, since our study included only those patients who were referred and diagnosed, it likely underestimates the effect of structural racism on access to therapy for youth of color [54]. Given the limited number of clinicians with competence in treating eating disorders, [26, 33, 34] capacity to deliver therapy in other languages is also generally limited. Finally, these findings are limited to treatment access as the primary outcome, in the absence of examining treatment outcomes (e.g., remission or symptom improvement).

## Conclusions

There are multiple structural barriers to equitable eating disorder care for adolescents and young adults. Publicly-insured youth with eating disorders have limited access to evidence-based psychological treatment. Youth of color are less likely to be diagnosed and receive recommended treatment, even after adjusting for demographic and clinical characteristics. Across socioeconomic and racial groups, patients who are hospitalized are more likely to receive appropriate care. While access to comprehensive mental health care is an important step towards equitable care, important changes are needed within our healthcare system. This includes addressing systemic inequities that contribute to disparities in care for youth of color and adopting a multidisciplinary approach to treatment to help patients access appropriate levels of care. Future research would benefit from understanding perceived barriers to care for youth of color or those with public insurance and their caregivers. Further, the field is tasked with understanding institutional policies and public health initiatives to make strides towards improved health equity for more diverse youth and those with public insurance.

## Supplementary Information

**Additional file 1:** Supplemental table S1 shows patients’ receipt of recommended treatment by diagnosis and treatment type.

## Data Availability

The datasets analyzed during the current study are not publicly available due to patient privacy concerns, but anonymized statistical results may be reasonably requested from the corresponding author.

## Abbreviations

AN: Anorexia nervosa
AAN: Atypical anorexia nervosa
ARFID: Avoidant restrictive food intake disorder
BED: Binge eating disorder
BN: Bulimia nervosa
CBT: Cognitive behavioral therapy
FBT: Family-based treatment
CHIP: Children’s Health Insurance Program
DSM-5: Diagnostic and Statistical Manual of Mental Disorders Fifth Edition
HR: Heart rate
IRB: Institutional Review Board
OSFED: Other Specified Feeding or Eating Disorder
SBP: Systolic blood pressure
UFED: Unspecified Feeding or Eating Disorder

## Footnotes

1 For the purposes of this paper, the terms people of color, youth of color, and children of color include the following racial and ethnic minorities within the United States: Latinx (also known as Hispanic or Latino), Asian, Black/African American, Native American/Alaskan Native, Native Hawaiian/Other Pacific Islander.

2 For the purposes of this paper, the following terms relate to US census definitions as follows. Latinx refers to “Hispanic or Latino”. Asian, Black/African American, Native American/Alaskan Native, Native Hawaiian/Other Pacific Islander, and White refer to those identifying by the corresponding race and not identifying as “Hispanic or Latino” ethnicity.
