# Supplementary material for: Disparities in access to eating disorders treatment for publicly-insured youth and youth of color: a retrospective cohort study
Source: J Eat Disord. 2023 Jan 24;11:10. doi: 10.1186/s40337-022-00730-7 (PMC9875472; doi:10.1186/s40337-022-00730-7)
Supplement: Supplementary file 1 — Additional file 1: Supplemental table S1 shows patients’ receipt of recommended treatment by diagnosis and treatment type. [file 40337_2022_730_MOESM1_ESM.docx]

Supplemental: Table S1. Receipt of recommended treatment by diagnosis and treatment type

|  |  | Received recommended treatment | | |  |
| --- | --- | --- | --- | --- | --- |
|  |  | Yes (*n* = 553, 60.6%) |  | No (*n* = 359, 39.4%) |  |
|  |  |  | *n (%)* |  | *n (%)* |
| AN |  | FBT/IFT CBT/DBT IOP/PHP/RTP None  Total | 204 (55.0%) 27 (7.3%) 32 (8.6%) 1 (0.3%)  264 (71.2%) | CBT/DBT Individual/Family None  Total | 4 (1.1%) 69 (18.6%) 34 (9.2%)  107 (28.8%) |
|  |  |  |  |  |  |
| AAN |  | FBT/IFT CBT/DBT IOP/PHP/RTP None  Total | 63 (48.5) 9 (6.9%) 11 (8.5%) 1 (0.8%)  84 (64.6%) | Individual/Family/Group None  Total | 31 (23.8%) 15 (11.5%)  46 (35.4%) |
|  |  |  |  |  |  |
| ARFID |  | FBT CBT ERP PHP Individual  Total | 22 (40.0%) 6 (10.9%) 1 (1.8%) 2 (3.6%) 1 (1.8%)  32 (58.2%) | Individual None  Total | 9 (16.4%) 14 (25.5%)  23 (41.8%) |
|  |  |  |  |  |  |
| BED |  | CBT/DBT RTP  Total | 6 (54.5%) 1 (9.1%)  7 (63.6%) | Individual  Total | 4 (36.4%)  4 (36.4%) |
|  |  |  |  |  |  |
| BN |  | FBT CBT/DBT IOP/PHP/RTP  Total | 9 (16.4%) 18 (32.7%) 10 (18.2%)  37 (67.3%) | Individual None  Total | 7 (12.7%) 11 (20.0%)  18 (32.7%) |
|  |  |  |  |  |  |
| OSFED |  | FBT/IFT CBT/DBT PHP Individual  Total | 11 (23.4%) 7 (14.9%) 2 (4.3%) 2 (4.3%)  22 (46.8%) | CBT Individual None  Total | 1 (2.1%) 8 (17.0%) 16 (34.0%)  25 (53.2%) |
|  |  |  |  |  |  |
| Rumination disorder |  | None | 1 (100%) |  |  |
|  |  |  |  |  |  |
| UFED |  | FBT CBT/DBT IOP/RTP Individual None  Total | 56 (23.8%) 17 (7.2%) 26 (11.1%) 3 (1.3%) 1 (0.4%)  103 (43.8%) | CBT/DBT Individual/Family None  Total | 4 (1.7%) 69 (29.4%) 59 (25.1%)  132 (56.2%) |
|  |  |  |  |  |  |
| Symptoms without ED diagnosis |  | Individual/Family | 3 (42.9%) | None | 4 (57.1%) |
| AN = anorexia nervosa; AAN = atypical anorexia nervosa; ARFID = avoidant restrictive food intake disorder; BED = binge eating disorder; BN = bulimia nervosa; OSFED = other specified feeding or eating disorder; UFED = unspecified feeding or eating disorder; ED = eating disorder.  FBT = family-based treatment; IFT = intensive family treatment (based in FBT); CBT = cognitive behavioral therapy; DBT = dialectical behavior therapy; IOP = intensive outpatient therapy; PHP = partial hospitalization; RTP = residential treatment program; ERP = exposure and response prevention.  Note: CBT for conditions other than disordered eating was coded as not indicated. In rare cases, other than continued medical monitoring, no further treatment was recommended (e.g., if the eating disorder was in full remission or in partial remission and improving). | | | | | |
